# The Potential Mechanism of Cannabidiol (CBD) Treatment of Epilepsy in Pentetrazol (PTZ) Kindling Mice Uncovered by Multi-Omics Analysis

**DOI:** 10.3390/molecules28062805

**Published:** 2023-03-20

**Authors:** Hongyuan Lu, Qinbiao Wang, Xiaowen Jiang, Yanyun Zhao, Miao He, Minjie Wei

**Affiliations:** 1 Department of Pharmacology, School of Pharmacy, China Medical University, Shenyang 110122, China; 2 School of Traditional Chinese Materia Medica, Shenyang Pharmaceutical University, Shenyang 110016, China

**Keywords:** CBD, seizures, transcriptome, metabolome, bioinformation, multi-omics

## Abstract

Cannabidiol (CBD) is the main active ingredient in the cannabis plant used for treating epilepsy and related diseases. However, how CBD ameliorates epilepsy and its effect on the hippocampus remains unknown. Herein, we evaluated how CBD ameliorates seizure degree in pentylenetetrazol (PTZ) induced epilepsy mice after being exposed to CBD (10 mg/kg p.o). In addition, transcriptome and metabolomic analysis were performed on the hippocampus. Our results suggested that CBD could alleviate PTZ-induced seizure, of which the NPTX2, Gprc5c, Lipg, and Stc2 genes were significantly down-regulated in mice after being exposed to PTZ. Transcriptome analysis showed 97 differently expressed genes (CBD) and the PTZ groups. Metabonomic analysis revealed that compared with the PTZ group, 41 up-regulated and 67 down-regulated metabolites were identified in the hippocampus of epileptic mice exposed to CBD. The correlation analysis for transcriptome and metabolome showed that (±) 15-HETE and carnitine C6:0 were at the core of the network and were involved in the positive or negative regulation of the related genes after being treated with CBD. In conclusion, CBD ameliorates epilepsy by acting on the metabolism, calcium signaling pathway, and tuberculosis pathways in the hippocampus. Our study provided a practical basis for the therapeutic potential of treating epilepsy using CBD.

## 1. Introduction

Epilepsy is one of the most common chronic neurological diseases, affecting about 70 million people worldwide [1]. Although around 20 antiepileptic drugs are available, about 20% of epilepsies are drug-resistant [2]. Treatment-resistant epilepsies greatly influence the cognitive behavior and quality of life of the affected patients. Therefore, new therapeutics for epilepsy treatment are urgently needed [3].

Cannabis has been cultivated for more than 6000 years. In the past decade, there has been a growing interest in cannabis-based therapy. δ-9-tetrahydrocannabinol (9-THC) and cannabidiol (CBD) are the richest and most common components in the Cannabis sativa plant in more than 100 cannabinoids [4]. THC is an endogenous cannabinoid of cannabis receptors CB1 (CB1Rs) and CB2 receptors (CB2Rs), but it is a partial (impure) agonist in pharmacology [5]. Recent evidence shows that activation of the CB1 receptor by THC can reduce the production of neuronal growth factors and affect other signal cascades involved in synapse formation. CBD ameliorates many neurological diseases, including Alzheimer’s disease, Parkinson’s disease, autistic migraine, multiple sclerosis, and epilepsy [6,7]. Cannabis has been used to treat epilepsy since ancient times. Recent case reports have shown that cannabis products could treat seizures [8]. A plant-derived, highly purified CBD formulation has been approved by the US Food and Drug Administration and European Medicines Agency for treating seizures associated with Dravet syndrome and Lennox–Gastaut syndrome [9].

CB1R and the endocannabinoid system are involved in several aspects of central nervous system activities and disorders [10]. The hippocampus is one of the most susceptible regions in the brain vulnerable to status epilepticus (SE)-induced injury [11]. The CB1 receptor is one of the most abundant G protein-coupled receptors in the central nervous system, and its concentration is exceptionally high in the hippocampus [12]. Several cannabinoid receptors are overexpressed in the hippocampus, including CA1, CA3, and the dentate gyrus [13]. Therefore, the affinity of CBD to cannabinoid receptors (CB1R and CB2R) is relatively low. It is believed that the antiepileptic activity of CBD does not only involve the endocannabinoid system [14]. Many studies have reported the protective effect of CBD on the hippocampus during seizures [15,16] and the regulation of equilibrative nucleoside transporter (ENT) transporters, GPR55 receptors, and the transient receptor potential cation channel subfamily M member 8 (TRPM8) channels involved in neuronal hyperexcitability by CBD [17]. CBD also regulates the activity of 5HT1A serotonergic receptors, glycine receptors, and TRPA1 channels, which helps regulate intracellular calcium concentration [18]. Although several hypotheses have been proposed for the mechanism of action of CBD against epilepsy, the exact mechanism is still unclear.

In the search for alternative epilepsy treatments, it is imperative to find an appropriate animal model for seizures. The kindling model induced by an overdose of pentylenetetrazol (PTZ) is one of the experimental models extensively used for inducing seizures [19]. In the present study, we analyzed the effect of CBD in the mice hippocampal tissue with PTZ-induced by integrating transcriptome and metabolomics analyses. The aim of this study was to investigate the molecular mechanism of PTZ-induced seizures and the protective mechanism of CBD on the hippocampus.

## 2. Results

### 2.1. CBD Attenuated the Latency of PTZ-Kindled Mice

**Figure 1:**
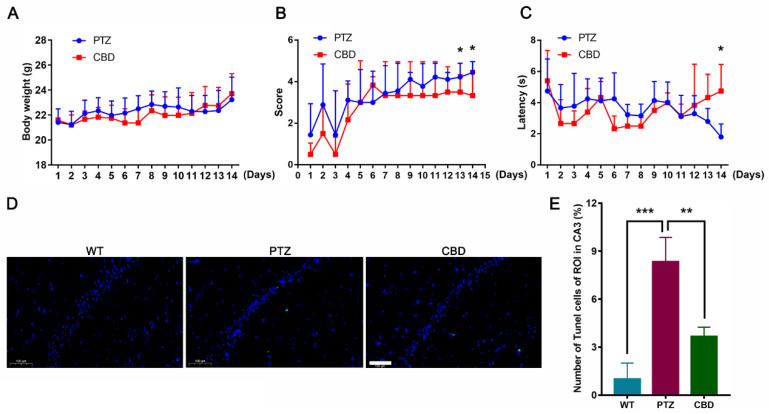
CBD attenuated the latency of PTZ-kindled mice. (**A**) The body weight of mice in the PTZ group and the CBD group, *n* = 10. (**B**) The seizure scores of mice in the PTZ group and the CBD group, *n* = 10. (**C**) The seizure latency of mice in the PTZ group and the CBD group, *n* = 10. (**D**) Representative TUNEL stained sections obtained from different groups of mice, *n* = 6. Scale bars = 100 µm. (**E**) Quantification of the TUNEL-positive number in hippocampus. * *p* < 0.05, ** *p* < 0.01, *** *p* < 0.001.

The kindling effect of PTZ on seizures were induced by intraperitoneal injection of 36 mg/kg/day PTZ after the mice fasted for 8 h. As illustrated in Figure 1A, the body weight of the PTZ group and the vehicle group did not change significantly. In the PTZ group, repeated administration of PTZ led to a gradual increase in seizure scores, eventually leading to generalized clonic tonic seizures (Figure 1B). Compared with the PTZ group, CBD (10 mg/kg, p.o) pretreatment significantly inhibited the progression of kindling at 13 and 14 days, which was manifested as a reduction in the seizure score. In addition, we also analyzed the latency of the first seizures. As shown in Figure 1C, CBD exposure can significantly increase the seizures latency of mice on day 14.

### 2.2. CBD Can Reverse Alkaline Phosphatase Deficiency in Epileptic Mice

**Figure 2:**
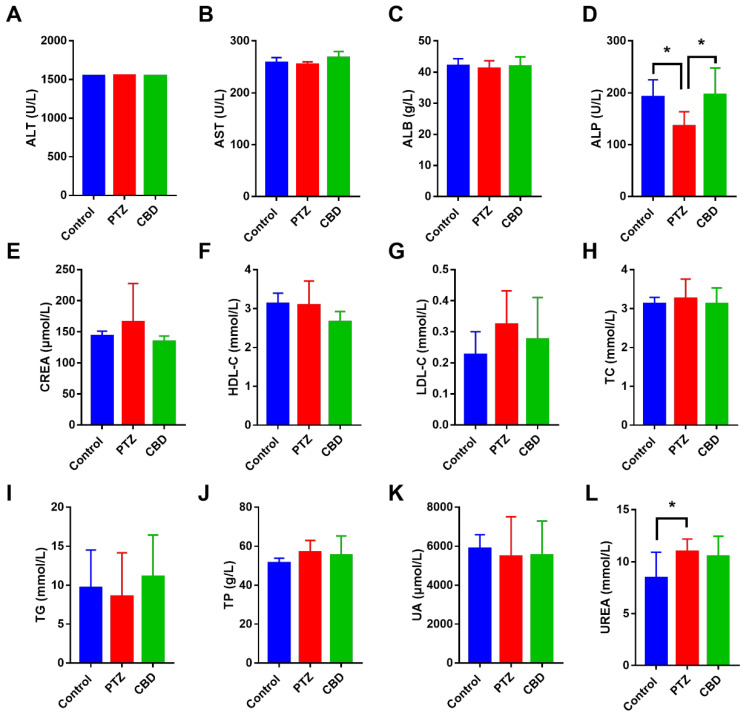
The blood biochemistry of mice in different groups. (**A**–**L**) The levels of glutamic-pyruvic transaminase (ALT), glutamic-oxaloacetic transaminase (AST), albumin (ALB), serum alkaline phosphatase (ALP), creatinine (CREA), high-density lipoprotein (HDL-C), low density lipoprotein (LDL-C), total cholesterol (TC), triglyceride (TG), total protein (TP), uric acid (UA), and urea in serum, *n* = 10. * *p* < 0.05.

In order to understand the changes in blood biochemistry of the mice in the seizure, as well as to explore the safety of CBD in mice at an oral dose, we performed a fully automated blood biochemical analysis on the peripheral blood of the mice. We explored the levels of ALT, AST, ALB, ALP, CRA, HDL-C, LDL-C, TC, TG, TP, UA, and UREA in the peripheral blood. The results showed that the ALP level of mice in the PTZ group was decreased significantly (*p* = 0.02) compared with that of the control group, while the ALP level in the CBD exposure group were significant decreased (*p* = 0.01). In addition, the level of UREA in the PTZ group was increased significantly compared with that of the control group (Figure 2).

### 2.3. Gene Expression Profiling Analysis of PTZ and CBD vs. PTZ

**Figure 3:**
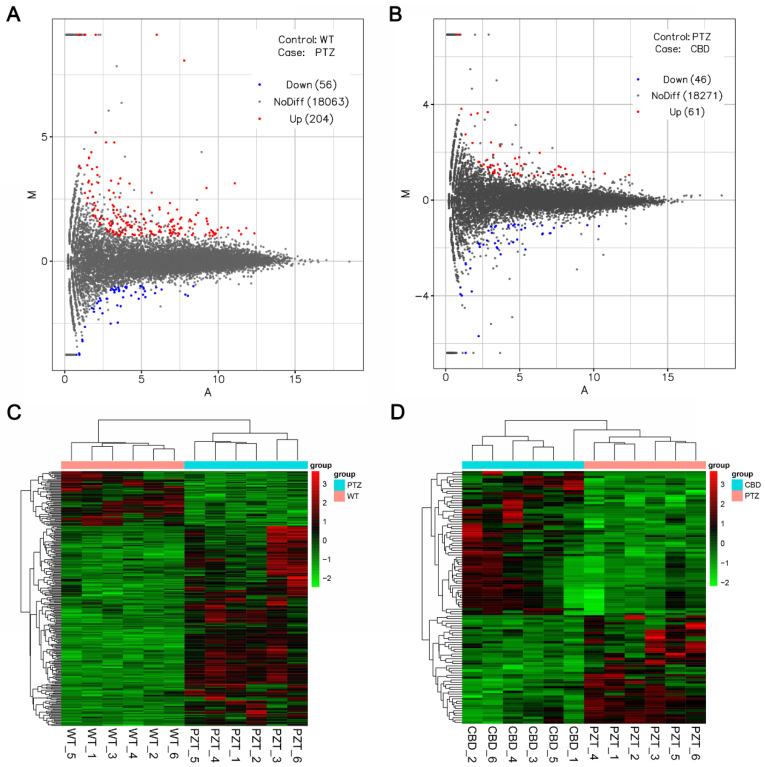
Gene expression profiling analysis of PTZ and CBD vs. PTZ. (**A**) The relationship between the different genes in each group. (**B**) The number of up-regulated and down-regulated DEGs between the groups. (**C**,**D**) The cluster analysis between different groups, *n* = 6 per group.

In order to clarify the mechanism of PTZ kindling seizures and the mechanism of CBD on seizures, we performed transcriptome sequencing analysis. Compared with the control group, 210 differential expression genes (DEGs) were up-regulated and 52 DEGs were down regulated in PTZ group (Figure 3A). Meanwhile, there were 45 DEGs up-regulated and 52 DEGs were down regulated in the CBD group compared with the PTZ group (Figure 3B). See Table S1 for details. Cluster analysis is used to judge the expression patterns of differentially expressed genes under different experimental conditions, so the unknown biological connections between genes can be found through expression clustering. As shown in Figure 3C, the up-regulated genes in the PTZ group were significantly more than the down-regulated genes, and showed good clustering. Meanwhile, the number of up- and down-regulated genes in CBD group is basically the same (Figure 3D).

### 2.4. The Key Pathway of PTZ Kindling Seizure on Hippocampus and the Hippocampal Protective Mechanism of CBD on Seizures

**Figure 4:**
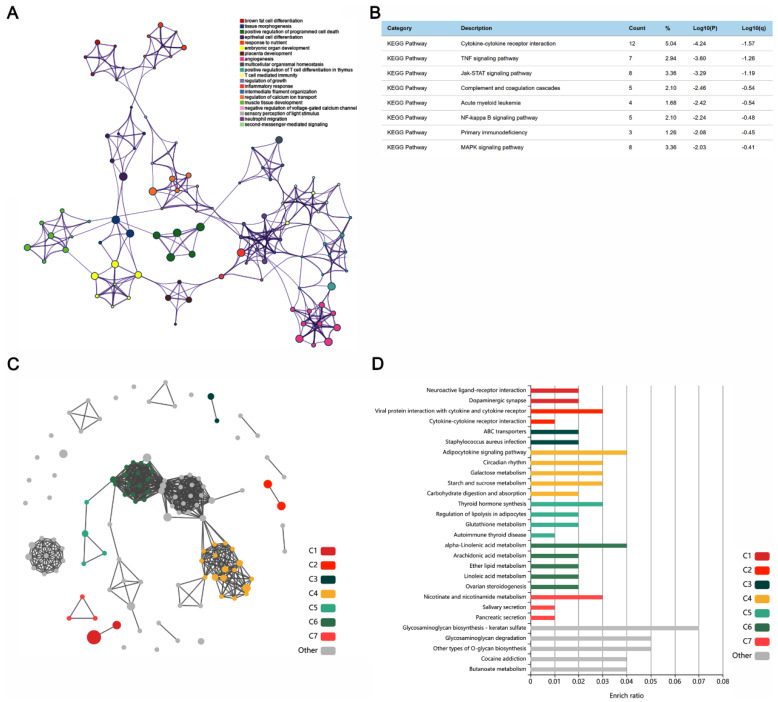
The GO network analysis and KEGG enrichment analysis on DEGs of the PTZ group and CBD group. (**A**) The GO network in the PTZ group. (**B**) The KEGG pathway analysis results in the PTZ group. (**C**) The network analysis on the KEGG signaling pathway by KOBAS. (**D**) The KEGG pathway analysis results in the CBD group.

In order to explore the key pathway of PTZ kindling seizure on hippocampus and the protective mechanism of CBD on hippocampus, we performed go network analysis and KEGG enrichment analysis on DEGs of PTZ group and CBD group, respectively. As illustrated in Figure 4A, the regulation of growth occupies the core position of the network in the PTZ group. This leads to the regulation of calcium transport, which leads to the response of the inflammatory factors. KEGG pathway analysis results showed that hippocampal inflammatory pathways were significantly activated after PTZ kindled epilepsy, such as the cytokine receptor interaction, TNF signaling pathway, Jak-STAT signaling pathway, and NF-κB signaling pathway (Figure 4B).

Next, we used KOBAS to conduct a network analysis on the KEGG signaling pathway. Linolenic acid and arachidene metabolic pathways and glycosaminoglycan biosynthesis and degradation pathways occupy the core position of the network (Figure 4C,D). These results suggest that the metabolic pathways in the hippocampus of epileptic mice after CBD exposure have played a key role, so next we conducted a metabolomics analysis.

### 2.5. Metabolite Profiling Analysis of the WT, PTZ, and CBD Group

**Figure 5:**
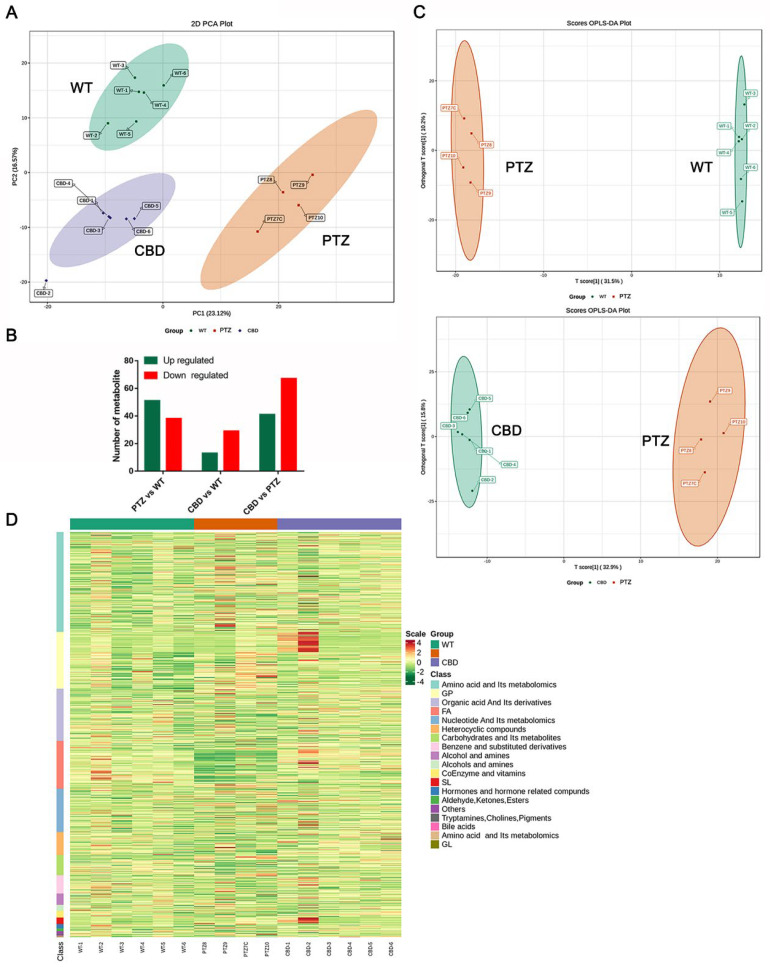
Metabolite profiling analysis of the WT, PTZ, and CBD group. (**A**) The PCA score scatter plot of the positive ionization mode. (**B**) The number of up-regulated and down-regulated metabolites between the groups. (**C**) The result of the Partial Least Squares-Discriminant Analysis (PLS−DA). The OPLS-DA score scatter plots were compared between the groups. (**D**) The clustering heat map analysis of all samples. *n* = 6 in the PTZ group, *n* = 4 in the CBD group.

To understand the overall metabolic difference between the samples and the variability between the samples in the group, we first conducted principal component analysis. The PCA score scatter plot of the positive ionization mode shows that there are obvious differences in the grouping between each group, and the degree of clustering within each group is higher (represented by the same-colored dots in the circle) (Figure 5A). Figure 5B illustrates that there were 51 up-regulated metabolites and 38 regulated metabolites in the PTZ group compared with the WT group. Meanwhile, compared with the PTZ group, 41 up-regulated metabolites and 67 down-regulated metabolites were found in the hippocampus of epileptic mice exposed to CBD. See Tables S2 and S3 for details. To identify discriminating metabolites and differentiate the two groups, we conducted Partial Least Squares-Discriminant Analysis (PLS-DA). As shown in Figure 5C, the PLS-DA model demonstrated clear separation of samples from the two groups. Figure 5D shows the clustering heat map analysis of all the samples, presenting the differences in the major metabolites of each sample.

**Figure 6:**
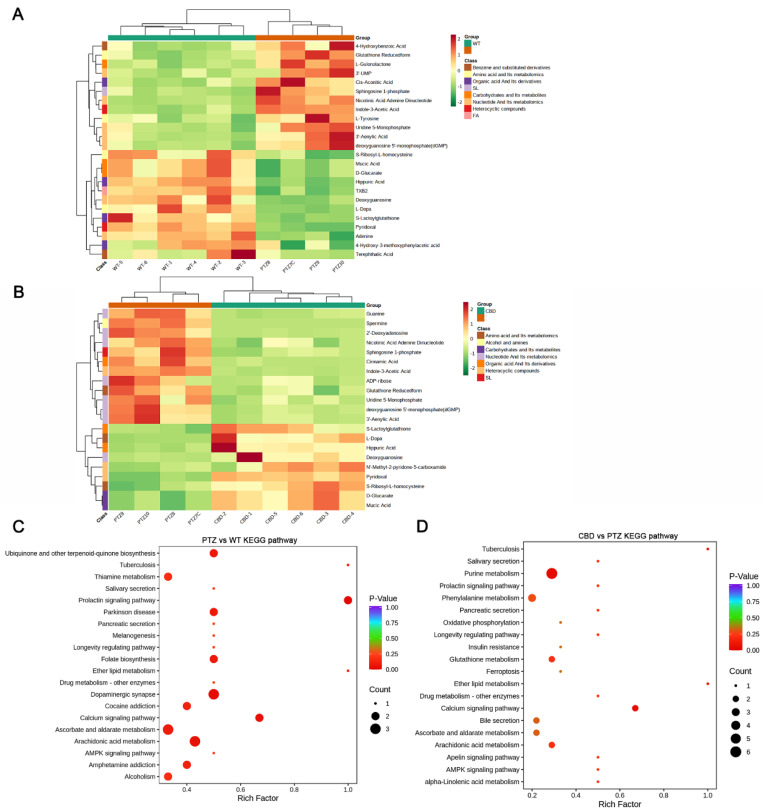
Functional analysis of pathways related to different metabolites in the PTZ and CBD group. The cluster analysis of the PTZ (**A**) and CBD (**B**) group. (**C**) The KEGG enrichment analysis showed the pathways of differential metabolite enrichment after PTZ kindled seizure. (**D**) The pathways of differential metabolite enrichment in hippocampus of PTZ kindled seizure mice treated with CBD.

To better understand the changes of substances in potentially important metabolic pathways in different groups, we analyzed the KEGG metabolic pathway that contains at least five differential metabolites and performed cluster analysis on the relative content of all differential metabolites in these pathways. As illustrated in Figure 6A, D-Glucarate, Deoxyguanosine, Hippuric Acid, L-Dopa Pyridoxal, Mucic Acid, and S-Ribosyl-L-homocysteine were significantly elevated in the hippocampus of mice with PTZ kindling, and CBD exposure can reverse this increase (Figure 6B).

KEGG enrichment analysis showed that the top three pathways of differential metabolite enrichment after PTZ kindled seizure were the prolactin signaling pathway, dopaminergic synapse, and calcium signaling pathway (Figure 6C). Meanwhile, the top three pathways of differential metabolite enrichment in the hippocampus of PTZ kindled seizure mice treated with CBD were purine metabolism, calcium signaling pathway, and tuberculosis (Figure 6D).

### 2.6. Metabolites Fuzzy Cluster Analysis of the WT, PTZ, and CBD Group

**Figure 7:**
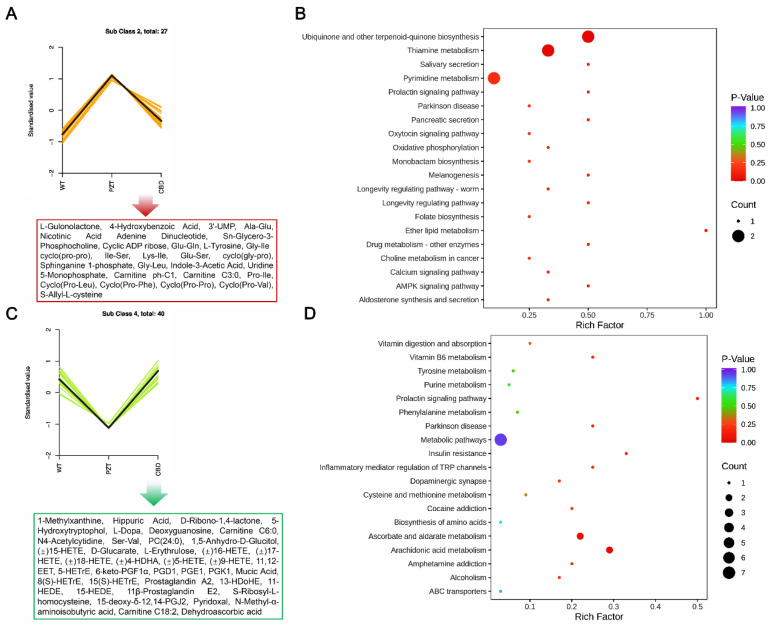
Metabolites fuzzy cluster analysis of the WT, PTZ, and CBD group. (**A**) The K-means clustering (K_means) analysis of 27 differential metabolites that were significantly increased in the PTZ group. (**B**) The KEGG pathway analysis showed the enrichment of the 27 differential metabolites. (**C**) The K-means clustering (K_means) analysis of 40 metabolites that were significantly up-regulated by the pre-exposure to CBD in the PTZ group. (**D**) The KEGG pathway analysis showed the enrichment of the 40 differential metabolites.

Furthermore, in order to investigate the change trend of the relative content of metabolites in different samples, the relative content of different metabolites was standardized, and then K-means clustering (K_means) analysis was performed. As illustrated in Figure 7A, a total of 27 differential metabolites were significantly increased in the PTZ group, while the WT group and the CBD group were significantly decreased. These metabolites may be up-regulated metabolites mediated by CBD reversal of PTZ. Therefore, we performed KEGG pathway analysis on these metabolites. Figure 7B shows that these metabolites were mainly enriched in Ubiquinone and other terpenoid-quinone biosynthesis, Thiamine metabolism, and Pyrimidine metabolism. Figure 7C illustrates that the pre-exposure to CBD significantly up-regulated 40 metabolites in the PTZ group, and these metabolites were mainly enriched in Ascorbate and aldarate metabolism and Arachidonic acid metabolism (Figure 7D).

### 2.7. Correlation Analysis between Transcriptome and Metabolome

**Figure 8:**
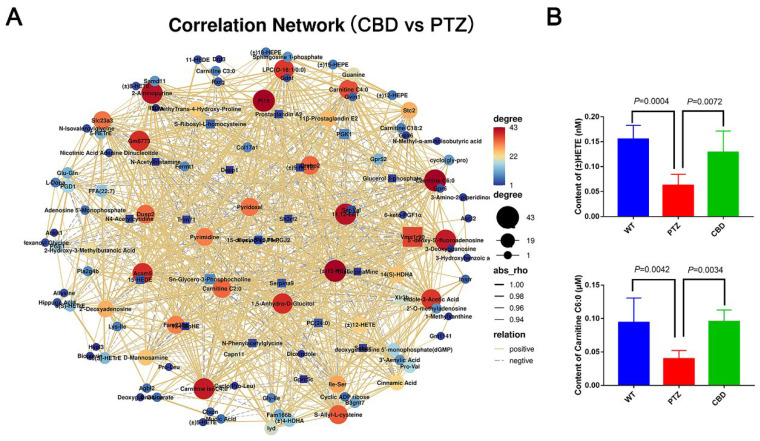
Correlation analysis of transcriptome and metabolome. (**A**) The correlation network between the CBD group and PTZ group. (**B**) The content of (+/−)15-HETE and carnitine C6:0 in hippocampus of mice in each group. *n* = 6, the error bar represents SD.

In order to understand the relationship between transcriptome differentially expressed genes and metabolome differentially expressed metabolites, we performed a correlation analysis of the two omics data. As shown in Figure S1, DEGs Nptx2, Gprc5c, Lipg, and Stc2 were the core nodes of the network and were positively correlated with numerous genes and metabolites, which indicated that these genes were involved in the regulation of many genes and metabolites in the hippocampus during PTZ kindled seizure. For metabolites, Hippuric acid, (±)-HETE and Carnitine C6:0 were the core nodes of the network at the core of the network and were involved in the positive or negative regulation of the related genes (Figure 8A). (±)15-HETE and Carnitine C6:0 levels were decreased in the hippocampus in the PTZ kindled mice, while CBD pretreatment could reverse the (±)15-HETE and Carnitine C6:0 levels in mice hippocampus (Figure 8B). These results revealed a causal relationship between CBD regulation of hippocampal transcription and metabolic levels in epileptic pathology.

## 3. Discussion

Epilepsy is a central nervous system disease, which is characterized by uncontrollable seizures in the upper or lower limbs. As the pathogenesis of epilepsy is extremely complicated, our current knowledge is not enough to fully understand epilepsy at the molecular level. The cannabis component monomer, CBD, has been approved by the FDA of the United States for treating seizures associated with Dravet Syndrome and Lennox–Gastaut Syndrome, characterized by different recurrent seizures related to mental retardation. However, the protective mechanism of CBD on the hippocampus during seizure remains unknown. Our study confirmed that CBD could ameliorate PTZ-kindled seizures. In addition, transcriptome and metabolome sequencing revealed that the critical role of inflammatory pathways were regulated by CBD in the hippocampus of PTZ-kindled mice, and CBD ameliorates epilepsy by mainly regulating the expression of (±)15-HETE, PI15, and carnitine C6:0 in the hippocampus.

Pre-clinical and clinical studies have shown that CBD has a neuroprotective role in treating epilepsy by multiple mechanisms: (1) it protects neuronal damage by inhibiting the release of Glu. Glu release is triggered by retrograde signaling in the synapses in the endocannabinoid system (ECS), and CBD is one of the cannabinoids that interact with ECS [20]. Five endocannabinoids that bind to cannabis receptors CB1 (CB1Rs) or cannabis receptors CB2 (CB2Rs) have been identified [21]. However, so far, only the two most related endocannabinoids, 2-arachidonoylglycerol (2-AG), and anandamide (AEA), play a relevant role in ECS function [22]; (2) CB1Rs and CB2Rs are partially activated by CBD, activating several signaling pathways, such as PKC, PI3K/Akt, and ERK pathways that promote the growth of neurites [23,24,25]. However, the further downstream effects of these signal pathways are complex and have not been fully explored.

Our study confirmed that CBD ameliorated PTZ-induced kindling but had no effect on the weight of the mice. Except for alkaline phosphotase (ALP) and urea, we found no significant changes in the blood biochemistry of PTZ-kindled mice. ALP deficiency or low phosphatase disorder affects the entry of pyridoxal 50-phosphate (PLP) into the brain, which is related to a seizure disorder [26]. However, we found no significant change in the serum ALP levels between the epileptic children taking levetiracetam and the control group (*n* = 23) or oxcarbazepine (*n* = 20) [27]. Our study found that CBD reduces ALP levels in peripheral blood for the first time. Previous studies have found that the urea level in the peripheral blood is significantly high in patients with epilepsy [28,29], which is consistent with our results. However, how this occurs has not been clarified.

PTZ interacts with the benzodiazepine recognition site of the gamma-aminobutyric acid receptor [30], and this involves changes in ion movement [31] but not the degradation of cyclic nucleotides [32]. However, the mechanism of PTZ-induced epilepsy remains unknown. The hippocampus produces epileptiform activities. The hippocampus exhibits epilepsy-related abnormalities, such as hippocampal hardening and mossy fiber sprouting [33]. The omics analysis of the hippocampus of epileptic rats helps in understanding the molecular mechanism of these pathological changes. Xu et al. found significant changes in the calcium signal pathway, neuroactive ligand-receptor interaction pathway, and NF-κB pathway in the hippocampus of PTZ-induced epileptic rats [34]. By sequencing the hippocampal transcriptome of PTZ-induced kindling mice, we identified many differentially expressed genes related to inflammatory pathways, including cytokine receptor interaction, TNF signaling pathway, JAK-STAT signaling pathway, and NF- κB signaling pathway. KEGG enrichment analysis showed that the neuroactive ligand-receptor interaction pathway and dopaminergic synaptic pathway were regulated by CBD, which is consistent with previous studies [34], and the metabolism-related pathway was at the core of the network. Based on these findings, we performed a metabolomic analysis of the hippocampus of epileptic mice.

At present, there is limited research on the brain metabolome of PTZ-induced epilepsy. Zhu et al. performed a metabolome study on the cortex of PTZ-induced epilepsy mice. They found that the primary differential metabolites of epileptic mice were enriched in glycerophospholipid metabolism, nicotinate and nicotinamide metabolism, alanine, aspartate, and glutamate metabolism, and pyruvate metabolism pathway [35]. Unfortunately, this study was not based on variable importance in projection (VIP) to screen out the metabolites of the hippocampus of epileptic mice. The VIP value indicates the strength of the influence of the difference between the corresponding metabolites in the classification and discrimination of the samples in each group in the model. It is generally believed that the metabolites with VIP ≥ 1 differ significantly. Our study demonstrated that the primary metabolite enrichment pathways in the hippocampus of PTZ kindled mice were the prolactin signaling pathway, dopaminergic synapse, and calcium signaling pathway. In contrast, the main metabolic pathways in CBD pre-exposed epileptic mice were enriched in pure metabolism, calcium signaling, and tuberculosis related pathways.

## 4. Materials and Methods

### 4.1. Animals and Treatment

Adult male C57BL/6 mice weighing 22 ± 2 g (2 months old) were purchased from Beijing HFK Biological Co., Ltd. (Beijing, China) and kept at a constant room temperature (25 ± 2 °C) and humidity (40–60%) under standard laboratory conditions. The mouse breeding room maintains a 12-h light/dark cycle, and ensures that the mice can eat and drink water freely. Animal experiments were conducted in accordance with the “Regulations on the Administration of Laboratory Animals” promulgated by the Ministry of Science and Technology of the People’s Republic of China (1988) No. 134 and were approved by the Ethics Committee of China Medical University.

Pentylenetetrazole (PTZ) has a purity of >95% and was purchased from Bidepharm (Shanghai, China). PTZ was dissolved in saline by injection at a dose of 36 mg/kg (i.p.). The purity of CDB > 90%, purchased from biopurify (Chengdu, China), CBD was dissolved in 5% DMSO and resuspended in 0.5% CMC-Na, and the oral dose was 10 mg/kg/day. Randomized grouping was used to divide C57BL/6 mice into 2 groups with 10 mice in each group. After the mice fasted for 8 h, CBD was administrated by gavage before intraperitoneal injection of PTZ. Then, behavioral observation was carried out at the same time, until the seizure of mice was kindled. The PTZ group was not exposed to CBD, and the control group was only given saline.

### 4.2. Seizure Score

The seizure score refers to previous reports in the literature [36]. The seizure classification standard is based on the Racine classification. Grade 0: No twitch response; Grade Ⅰ: myoclonic jerks, facial twitches, and chewing; Grade Ⅱ: neck muscle spasm manifested as nodding with or without tail flick; Grade Ⅲ: one forelimb lifted with clonic; Grade Ⅳ: Both forelimbs are raised with clonus; Grade V: The whole body has clonus and even jumps and falls.

### 4.3. Serum Biochemical Analysis

On the second day after the mice successfully kindled, the mouse blood samples were collected by extracting the eyeball. The blood sample was centrifuged at 3000 rpm at 4 °C for 10 min, and the resulting serum was stored at −80 °C. The blood biochemical analyzer (Mindray, BS-120) was used to detect various biochemical indicators.

### 4.4. TUNEL Assay

The mice were sacrificed by cervical dislocation. The brains were removed immediately and placed into a solution that containing 4% paraformaldehyde in PBS, and then processed for paraffin embedding. TUNEL staining was carried out as previously described [37]. The dewaxed slices were hydrated and incubated with protease-K (1:9) at room temperature (RT) for 15 min. The slices were incubated with 0.1% triton for 20 min. After washing, they were mixed with a proper amount of buffer (TDT enzyme, dUTP, buffer at the ratio of 1:5:50), incubated at 37 °C for 2 h, and kept moist. The slices were washed with PBS for 3 times, 5 min each time. After PBS is removed, the DAPI dye solution was added and incubated for 10 min at room temperature in the dark. After the slices were washed with PBS, anti-fluorescence quenching sealing tablets were added to seal the slices. The slices were observed under a fluorescence microscope (Nikon Ni-E, Tokyo, Japan) and the images were collected. The ultraviolet excitation wavelength of DAPI was 330–380 nm, and the emission wavelength was 420 nm. The excitation wavelength of FITC was 465–495 nm and the emission wavelength was 515–555 nm. The sections of hippocampal CA1, CA2, CA3, and dentate gyrus (DG) were photographed and analyzed. For TUNEL positive cells, the positive apoptotic nuclei were green, and the average number of positive cells in the sample were calculated.

### 4.5. RNA Extraction and Analysis

Trizol reagent (Invitrogen Life Technologies, Waltham, MA, USA) was used to isolate total RNA, and then NanoDrop (Thermo, Waltham, MA, USA) was used to determine RNA concentration, quality, and integrity. We used 3μgRNA to build a sequencing library according to the following steps. First, we used poly-T oligosaccharide-linked magnetic beads to purify mRNA from total RNA. In Illumina’s proprietary lysis buffer, divalent cations are used for lysis at high temperatures. We used random oligonucleotides and Super Script II to synthesize first-strand cDNA. Subsequently, DNA polymerase I and RNase H were used for second-strand cDNA synthesis. In order to select cDNA fragments of 400–500 bp in length, the library fragments were purified using the AMPure XP system (Beckman Coulter). The DNA fragments with linker molecules at both ends were subjected to a 15-cycle PCR reaction using the Illumina PCR primer mix. The product was purified (AMPure XP system) and quantified using Agilent’s high-sensitivity DNA analysis on the Bioanalyzer 2100 system (Agilent). The sequencing library was then sequenced on the NovaSeq 6000 platform (Illumina) by Shanghai Personal Biotechnology Co., Ltd. (Shanghai, China).

### 4.6. Metabolites and Analysis

After the tissue was homogenized, it was freeze-thawed repeatedly, and then aspirated the supernatant for mass spectrometry analysis. The data acquisition instrument system mainly includes ultra high performance liquid chromatography (UPLC) (ExionLC AD) and tandem mass spectrometry (QTRAP, MS/MS). The liquid phase conditions include: chromatographic column (Waters ACQUITY UPLC HSS T3 C18 1.8 μm, 2.1 mm × 100 mm), mobile phase (A is ultrapure water with 0.1% formic acid, B is 0.1% acetonitrile: 0 min, 95%A; 11 min, 10%B; 12 min, 10%B; 12.1 min, 95%B; 14 min, 95%B), the flow rate is 0.4 mL/min, the column temperature is 40 °C, and the injection volume is 2 μL. Mass spectrometry acquisition conditions: Electrospray ion source (ESI) temperature 500 °C, mass spectrometer voltage 5500 V (positive), −4500 V (negative), ion source gas I (GSI) 55 psi, curtain gas (CUR) 25 psi, and the CAD parameters are set to high. In Qtrap, each ion pair is scanned based on the optimized declustering voltage (DP) and collision energy (CE).

### 4.7. Bioinformatics Analysis

#### 4.7.1. RNA-Seq Analysis

The raw data in fastq format was first processed by the perl script to remove the linker sequence and filter out low quality (low quality, the number of bases with a base quality value less than or equal to 25 accounts for more than 60% of the total reads) and N (N means that the base cannot be determined (base information) reads with a ratio greater than 5%, so as to get clean reads that can be used for subsequent analysis. We used HISAT2 software to compare the clean reads with the reference genome to obtain mapped reads for the subsequent analysis. FeatureCounts was used to calculate the number of reads compared to each gene, and to calculate the expected number of fragments per kilobase of transcript sequence per millions base pairs sequenced (FPKM) value of each gene according to the length of the gene. DESeq2 (http://bioconductor.org/packages/release/bioc/html/DESeq2.html) was used to analyze the differential expression of genes (DEGs) between groups. The default screening threshold for differentially expressed genes was |log_2_FC|> 1 and Padj < 0.05.

#### 4.7.2. Screening and Analysis of Differential Metabolites

The mass spectrometry data were processed by Analyst 1.63, and the metabolites of the sample were analyzed qualitatively and quantitatively by mass spectrometry based on the local metabolic database. The combination of fold change and VIP value of OPLS-DA model was used to screen differential metabolites. The metabolites with fold change ≥2 and fold change ≤0.5 were selected, and the metabolites with VIP ≥ 1 were significantly different.

### 4.8. Statistics

All statistical analyses in this study were performed using GraphPad Prism (version 7) software and RStudio. One-way ANOVA analysis was followed by Tukey post hoc analyses performed in all statistical analyses. Where appropriate, the difference was assessed by an unpaired *t*-test of variance. *p* < 0.05 was considered to be statistically significant.

## 5. Conclusions

In conclusion, our study showed that CBD can alleviate the symptoms of PTZ kindled epileptic mice. The results of transcriptome sequencing showed that the differential genes in the hippocampus of epileptic mice pre exposed to CBD were enriched in multiple metabolic pathways. Metabolomic analysis suggested that the metabolism, calcium signaling pathway, and tuberculosis pathway were involved in the improvement of the hippocampal function of epileptic mice induced by CBD. The combined analysis of transcriptomics and metabolomics showed that NPTX2, Gprc5c, Lipg, and Stc2 were the core regulatory genes of the network in the hippocampus of PTZ kindled mice, while (±) 15-HETE, PI15, and carnitine C6:0 were the core metabolites for CBD to improve the hippocampus of epileptic mice. A shortcoming of this research is that no loss and gain of the function assay were designed to verify the results above, and this needs to be followed up by future research.

## Data Availability

The datasets generated and/or analyzed during the current study are available from the corresponding author upon reasonable request.

## Supplementary Materials

The following supporting information can be downloaded at: https://www.mdpi.com/article/10.3390/molecules28062805/s1, Table S1: DEGs of PTZ group of WT group; Table S2: Differential metabolites between PTZ group and WT group; Table S3 Differential metabolites between CBD group and PTZ group; Figure S1: The correlation network between PTZ group and WT group; Table S1: DEGs of PTZ group of WT group; Table S2: Differential metabolites between PTZ group and WT group.
