# Supplementary material for: The Potential Mechanism of Cannabidiol (CBD) Treatment of Epilepsy in Pentetrazol (PTZ) Kindling Mice Uncovered by Multi-Omics Analysis
Source: Molecules. 2023 Mar 20;28(6):2805. doi: 10.3390/molecules28062805 (PMC10056192; doi:10.3390/molecules28062805)
Supplement: Supplementary file 1 [file molecules-28-02805-s001.zip › molecules-2224450-supplementary.pdf]

Table S1. DEGs of PTZ group of WT group.

Group	Up-regulated DEGs	Down-regulated DEGs
PTZ vs WT	Nptx2, Zbtb1, Sertad1, Bcor, Ccnf, Fkbp5, Igsf9, Stc2, Lipg, Hif3a, Dpm3, Glce, Maff, Gprc5c, Galnt15, Baz1a, Trip10, Il1r1, Drd4, Gadd45b, Cdkn1a, Tgfb1i1, Rgs2, Cebpa, Ascl2, B3gnt7, Fosb, Sh2b2, Crybg2, Metrnl, Efna1, Pim1, Ptgs2, Egr3, Lsm11, Map3k6, Ppp1r13l, Htra4, Inhba, Bdnf, Nod2, Sh3bp1, Serpine1, Gem, Osmr, Etv3l, Tamalin	Frzb, Baiap2l2, Tnfsf10, Gask1b, Impg2, Tcap, Arr3, Npas4, H2bc6, Chp2, Cort, Olfr464, Edar, Abi3bp, Sebox, Cd80, Alox12, Espnl, Olfr78, B4galnt3, Ifit1bl2, Hjv, Fxyd4, Vmn1r90, Ccdc63, Fam71f2, Ptch2, Trim72, Phf11d, Cacna1s, Lrrc39, Bnipl, Ddias, Ccl19, Grhl2, Gdnf, Fbxo47, Nat8f5, Rab19, Vkorc1, Gpx6, Gm1141, Prrg4, Exo1, Tbx22, Aldh1a3, Olfr31, Alpk3
CBD vs PTZ	Agbl2, Tbx22, Crabp2, Derl3, Cacna1s, Mmp23, Clec12a, Depp1, Igfbpl1, Sebox, Drd3, Clspn, Ddias, Slc2a9, Dsg1c, Il20ra, Esco2, Alx3, Serpina9, Vmn1r90, Gvin1, Prkag3, Acsm5, Mstn, Pi15, Sh3rf2, Sun3, Gdnf, Ccdc63, Alpk3, Col17a1, Rxrg, Chaf1b, Gpr52, Brms1, Gpx6, Ndc80, Ppef2, Gpr6, Slco5a1, Insrr, Ankk1, Clec12b	Stc2, Gprc5c, Baz1a, B3gnt7, Efna1, Ascl2, Defb1, Samd11, Nkpd1, Trim71, Fermt1, Fam221a, Dusp2, Cldn23, Slc23a3, Il18r1, Krt6a, Drd4, Fam166b, Pla2g4b, Capn11, Rxfp3, Npw, Xlr3b, Iyd, Pcdh8, Ifi202b, Tmem171, Gxylt2, Pitx2, Zfp750, Calcb, Hyal3

Table S2. Differential metabolites between PTZ group and WT group.

Index	Formula	Compounds	Class I	VIP	Log2FC	Type
MADN0027	C6H6N4O2	1-Methylxanthine	Nucleotide And Its metabolomics	1.63	1.66	up
MADN0031	C5H5N5	Adenine	Nucleotide And Its metabolomics	1.57	1.52	up
MADN0091	C9H9NO3	Hippuric Acid	Organic acid And Its derivatives	1.70	1.74	up
MADN0113	C9H11N5O3	Biopterin	Heterocyclic compounds	1.63	1.60	up
MADN0230	C5H8O5	D-Ribono-1,4-lactone	Carbohydrates and Its metabolites	1.78	14.36	up
MADN0279	C10H11NO2	5-Hydroxytryptophol	Heterocyclic compounds	1.67	1.88	up
MADN0432	C9H11NO4	L-Dopa	Amino acid and Its metabolomics	1.33	3.94	up
MADP0068	C10H13N5O4	Deoxyguanosine	Nucleotide And Its metabolomics	1.60	1.14	up
MADP0119	C4H9NO2S	Methylcysteine	Amino acid and Its metabolomics	1.56	1.46	up
MADP0191	C13H21N3O8S	S-Lactoylglutathione	Organic acid And Its derivatives	1.67	2.66	up
MADP0264	C8H7NO2	Dioxindole	Heterocyclic compounds	1.69	2.24	up
MADP0267	C9H17NO4	Carnitine C2:0	FA	1.71	1.28	up
MADP0283	C7H11N3O	N-Acetylhistamine	Alcohols and amines	1.15	1.75	up
MADP0307	C9H10O3	3,4-Dimethoxybenzaldehyde	Benzene and substituted derivatives	1.34	1.17	up
MADP0464	C32H66NO7P	PC(24:0)	GP	1.69	2.10	up
MEDN0086	C8H6O4	Terephthalic Acid	Benzene and substituted derivatives	1.09	1.48	up
MEDN0210	C6H12O5	1,5-Anhydro-D-Glucitol	Carbohydrates and Its metabolites	1.75	3.39	up
MEDN0319	C9H10O4	4-Hydroxy-3-	Organic acid And Its derivatives	1.09	1.04	up

		methoxyphenylacetic acid				
MEDN0348	C20H34O6	TXB2	FA	1.64	1.35	up
MEDN0350	C20H32O3	(±)15-HETE	FA	1.67	1.41	up
MEDN0531	C6H10O8	D-Glucarate	Carbohydrates and Its metabolites	1.54	1.07	up
MEDN0753	C20H32O3	(±)16-HETE	FA	1.57	4.02	up
MEDN0755	C20H32O3	(±)17-HETE	FA	1.57	4.02	up
MEDN0757	C20H32O3	(±)18-HETE	FA	1.57	4.02	up
MEDN0758	C22H32O3	(±)4-HDHA	FA	1.68	1.64	up
MEDN0760	C20H32O3	(±)5-HETE	FA	1.69	1.50	up
MEDN0763	C20H32O3	(±)9-HETE	FA	1.69	1.50	up
MEDN0765	C20H32O3	11,12-EET	FA	1.69	1.13	up
MEDN0769	C22H32O3	14(S)-HDHA	FA	1.54	1.70	up
MEDN0779	C20H34O3	5-HETrE	FA	1.49	1.18	up
MEDN0781	C20H34O6	6-keto-PGF1α	FA	1.65	1.60	up
MEDN0791	C20H34O5	PGD1	FA	1.58	1.04	up
MEDN0792	C20H34O5	PGE1	FA	1.58	1.04	up
MEDN0798	C20H32O5	PGK1	FA	1.42	1.44	up
MEDN1314	C6H10O8	Mucic Acid	Carbohydrates and Its metabolites	1.54	1.07	up
MEDN1414	C17H28O3	12-HHT	FA	1.71	1.23	up
MEDN1419	C20H30O3	12-OxoETE	FA	1.77	9.25	up
MEDN1422	C20H34O3	8(S)-HETrE	FA	1.49	1.18	up
MEDN1423	C20H34O3	15(S)-HETrE	FA	1.49	1.18	up
MEDN1430	C20H30O4	Prostaglandin A2	FA	1.63	1.85	up
MEDN1446	C20H36O3	11-HEDE	FA	1.72	1.40	up
MEDN1447	C20H36O3	15-HEDE	FA	1.72	1.40	up
MEDN1452	C20H32O5	11β-Prostaglandin	FA	1.54	1.06	up

		E2				
MEDN1684	C10H20N2O4	S-Ribosyl-L-homocysteine	Amino acid and Its metabolomics	1.46	1.05	up
MEDP0530	C20H28O3	15-deoxy-δ-12,14-PGJ2	FA	1.68	1.97	up
MEDP0898	C8H9NO3	Pyridoxal	Heterocyclic compounds	1.71	2.41	up
MEDP1177	C5H11NO2	N-Methyl-α-aminoisobutyric acid	Amino acid and Its metabolomics	1.58	1.26	up
MEDP1381	C27H49NO4	Carnitine C20:2	FA	1.68	1.39	up
MEDP1386	C25H45NO5	Carnitine C18:2-OH	FA	1.57	1.29	up
MEDP1391	C25H45NO4	Carnitine C18:2	FA	1.67	1.41	up
MEDP2024	C6H6O6	Dehydroascorbic acid	Organic acid And Its derivatives	1.14	1.12	up
MADN0042	C6H6O6	Cis-Aconitic Acid	Organic acid And Its derivatives	1.57	-1.55	down
MADN0065	C6H10O6	L-Gulonolactone	Carbohydrates and Its metabolites	1.65	-1.08	down
MADN0235	C7H6O3	4-Hydroxybenzoic Acid	Benzene and substituted derivatives	1.44	-1.35	down
MADN0343	C9H13N2O9P	3'-UMP	Nucleotide And Its metabolomics	1.43	-1.06	down
MADN0439	C8H14N2O5	Ala-Glu	Amino acid and Its metabolomics	1.69	-2.00	down
MADP0072	C21H27N7O14P2	Nicotinic Acid Adenine Dinucleotide	Nucleotide And Its metabolomics	1.74	-1.84	down
MADP0142	C10H14N5O7P	Adenosine 5'-Monophosphate	Nucleotide And Its metabolomics	1.46	-1.03	down
MADP0182	C6H13NO5	D-Mannosamine	Alcohols and amines	1.54	-1.06	down
MADP0184	C10H14N5O7	3'-Aenylic Acid	Nucleotide And Its metabolomics	1.50	-1.10	down

	P					
MADP0196	C8H20NO6P	Sn-Glycero-3-Phosphocholine	Tryptamines,Cholines,Pigments	1.66	-1.83	down
MADP0206	C15H21N5O13P2	Cyclic ADP ribose	Nucleotide And Its metabolomics	1.75	-2.07	down
MADP0209	C10H17N3O6	Glu-Gln	Amino acid and Its metabolomics	1.71	-3.42	down
MADP0220	C10H17N3O6S	Glutathione Reducedform	Amino acid and Its metabolomics	1.60	-1.00	down
MADP0259	C9H11NO3	L-Tyrosine	Amino acid and Its metabolomics	1.37	-1.18	down
MADP0268	C11H21NO4	Carnitine isoC4:0	FA	1.73	-1.17	down
MADP0343	C8H16N2O3	Gly-Ile	Amino acid and Its metabolomics	1.53	-1.05	down
MADP0385	C10H14N2O2	cyclo(pro-pro)	Amino acid and Its metabolomics	1.37	-1.76	down
MADP0394	C9H18N2O4	Ile-Ser	Amino acid and Its metabolomics	1.72	-2.33	down
MADP0398	C12H25N3O3	Lys-Ile	Amino acid and Its metabolomics	1.63	-1.84	down
MADP0405	C8H14N2O6	Glu-Ser	Amino acid and Its metabolomics	1.55	-1.07	down
MADP0422	C7H10N2O2	cyclo(gly-pro)	Amino acid and Its metabolomics	1.16	-4.48	down
MEDN0588	C3H9O6P	Glucerol 3-phosphate	Organic acid And Its derivatives	1.42	-1.05	down
MEDN0602	C10H14N5O7P	deoxyguanosine 5'-monophosphate(dGMP)	Nucleotide And Its metabolomics	1.57	-1.17	down

MEDN1083	C18H38NO5P	Sphingosine 1-phosphate	SL	1.61	-1.15	down
MEDN1172	C18H40NO5P	Sphinganine 1-phosphate	SL	1.59	-1.15	down
MEDP0071	C8H16N2O3	Gly-Leu	Amino acid and Its metabolomics	1.52	-1.04	down
MEDP0275	C10H9NO2	Indole-3-Acetic Acid	Heterocyclic compounds	1.68	-2.05	down
MEDP0372	C9H13N2O9P	Uridine 5-Monophosphate	Nucleotide And Its metabolomics	1.43	-1.16	down
MEDP1289	C13H25NO4	Carnitine C6:0	FA	1.70	-1.12	down
MEDP1433	C14H19NO4	Carnitine ph-C1	FA	1.20	-1.12	down
MEDP1442	C11H21NO4	Carnitine C4:0	FA	1.75	-1.31	down
MEDP1665	C10H19NO4	Carnitine C3:0	FA	1.67	-2.26	down
MEDP1885	C11H20N2O3	Pro-Ile	Amino acid and Its metabolomics	1.17	-1.22	down
MEDP1919	C11H18N2O2	Cyclo(Pro-Leu)	Amino acid and Its metabolomics	1.13	-1.17	down
MEDP1926	C14H16N2O2	Cyclo(Pro-Phe)	Amino acid and Its metabolomics	1.15	-1.23	down
MEDP1927	C10H14N2O2	Cyclo(Pro-Pro)	Amino acid and Its metabolomics	1.33	-1.43	down
MEDP1928	C10H16N2O2	Cyclo(Pro-Val)	Amino acid and Its metabolomics	1.31	-1.40	down
MEDP1966	C6H11NO2S	S-Allyl-L-cysteine	Amino acid and Its metabolomics	1.68	-1.28	down

Table 3. Differential metabolites between CBD group and PTZ group.

Index	Formula	Compounds	Class I	VIP	p_value	Log2FC	Type
MADN0053	C4H6O4	D-Erythronolactone	Carbohydrates and Its metabolites	1.48	0.01	-1.01	up
MADN0128	C5H5N5O	Guanine	Nucleotide And Its metabolomics	1.66	0.01	-1.60	up
MADN0166	C6H10O5	1,6-anhydro-β-D-glucose	Carbohydrates and Its metabolites	1.27	0.04	-1.01	up
MADN0206	C5H10O3	2-Hydroxy-3-Methylbutanoic Acid	Organic acid And Its derivatives	1.57	0.00	-1.02	up
MADP0072	C21H27N7O14P2	Nicotinic Acid Adenine Dinucleotide	Nucleotide And Its metabolomics	1.44	0.00	-1.26	up
MADP0106	C10H13N5O3	2'-Deoxyadenosine	Nucleotide And Its metabolomics	1.70	0.01	-3.24	up
MADP0142	C10H14N5O7P	Adenosine 5'-Monophosphate	Nucleotide And Its metabolomics	1.59	0.03	-1.52	up
MADP0182	C6H13NO5	D-Mannosamine	Alcohols and amines	1.66	0.00	-1.96	up
MADP0184	C10H14N5O7P	3'-Aenylic Acid	Nucleotide And Its metabolomics	1.59	0.02	-1.52	up
MADP0196	C8H20NO6P	Sn-Glycero-3-Phosphocholine	Tryptamines,Cholines,Pigments	1.61	0.03	-1.72	up
MADP0206	C15H21N5O13P2	Cyclic ADP ribose	Nucleotide And Its metabolomics	1.52	0.00	-1.50	up
MADP0209	C10H17N3O6	Glu-Gln	Amino acid and Its metabolomics	1.69	0.03	-2.79	up
MADP0220	C10H17N3O6S	Glutathione Reducedform	Amino acid and Its metabolomics	1.47	0.00	-1.17	up
MADP0268	C11H21NO4	Carnitine isoC4:0	FA	1.72	0.00	-1.83	up
MADP0343	C8H16N2O3	Gly-Ile	Amino acid and Its	1.45	0.00	-1.04	up

			metabolomics				
MADP0382	C11H20N2O3	Pro-Leu	Amino acid and Its metabolomics	1.33	0.02	-1.08	up
MADP0394	C9H18N2O4	Ile-Ser	Amino acid and Its metabolomics	1.64	0.00	-2.30	up
MADP0398	C12H25N3O3	Lys-Ile	Amino acid and Its metabolomics	1.53	0.04	-1.56	up
MADP0422	C7H10N2O2	cyclo(gly-pro)	Amino acid and Its metabolomics	1.33	0.01	-1.66	up
MEDN0487	C6H11NO3	Allysine	Aldehyde,Ketones,Esters	1.50	0.00	-1.13	up
MEDN0537	C15H23N5O14P2	ADP-ribose	Nucleotide And Its metabolomics	1.26	0.08	-1.38	up
MEDN0588	C3H9O6P	Glucerol 3-phosphate	Organic acid And Its derivatives	1.44	0.06	-1.22	up
MEDN0602	C10H14N5O7P	deoxyguanosine 5'-monophosphate(dGMP)	Nucleotide And Its metabolomics	1.64	0.01	-1.54	up
MEDN1083	C18H38NO5P	Sphingosine 1-phosphate	SL	1.52	0.01	-1.45	up
MEDP0275	C10H9NO2	Indole-3-Acetic Acid	Heterocyclic compounds	1.73	0.00	-1.88	up
MEDP0332	C9H8O2	Cinnamic Acid	Organic acid And Its derivatives	1.67	0.01	-3.86	up
MEDP0372	C9H13N2O9P	Uridine 5-Monophosphate	Nucleotide And Its metabolomics	1.45	0.02	-1.06	up
MEDP1077	C11H15N5O4	2'-O-methyladenosine	Nucleotide And Its metabolomics	1.59	0.03	-1.43	up
MEDP1202	C10H12FN5O3	5'-deoxy-5'-fluoroadenosine	Nucleotide And Its metabolomics	1.63	0.00	-3.78	up
MEDP1289	C13H25NO4	Carnitine C6:0	FA	1.72	0.00	-1.66	up
MEDP1442	C11H21NO4	Carnitine C4:0	FA	1.71	0.00	-1.77	up
MEDP1658	C26H54NO6P	LPC(O-18:1/0:0)	GP	1.67	0.00	-1.11	up

MEDP1665	C10H19NO4	Carnitine C3:0	FA	1.64	0.01	-2.26	up
MEDP1737	C21H32O2	5α-Pregnane-3,20-dione	CoEnzyme and vitamins	1.13	0.16	-1.58	up
MEDP1838	C10H26N4	Spermine	Alcohol and amines	1.74	0.00	-13.39	up
MEDP1886	C10H18N2O3	Pro-Val	Amino acid and Its metabolomics	1.54	0.00	-1.03	up
MEDP1919	C11H18N2O2	Cyclo(Pro-Leu)	Amino acid and Its metabolomics	1.05	0.20	-1.02	up
MEDP1926	C14H16N2O2	Cyclo(Pro-Phe)	Amino acid and Its metabolomics	1.20	0.15	-1.02	up
MEDP1956	C5H5N5	2-Aminopurine	Nucleotide And Its metabolomics	1.63	0.00	-3.99	up
MEDP1966	C6H11NO2S	S-Allyl-L-cysteine	Amino acid and Its metabolomics	1.69	0.00	-1.19	up
MEDP2010	C8H11NO2	p-Hydroxyphenylethanolamine	Alcohol and amines	1.01	0.20	-4.68	up
MADN0017	C7H13NO3	N-Isovaleroylglycine	Amino acid and Its metabolomics	1.49	0.02	2.57	down
MADN0027	C6H6N4O2	1-Methylxanthine	Nucleotide And Its metabolomics	1.59	0.00	1.51	down
MADN0091	C9H9NO3	Hippuric Acid	Organic acid And Its derivatives	1.55	0.01	1.87	down
MADN0113	C9H11N5O3	Biopterin	Heterocyclic compounds	1.40	0.01	1.10	down
MADN0218	C10H11NO3	N-Phenylacetylglycine	Amino acid and Its metabolomics	1.38	0.06	2.49	down
MADN0230	C5H8O5	D-Ribono-1,4-lactone	Carbohydrates and Its metabolites	1.74	0.00	14.51	down
MADN0279	C10H11NO2	5-Hydroxytryptophol	Heterocyclic compounds	1.33	0.06	1.85	down
MADN0307	C7H6O3	3-Hydroxybenzoic acid	Benzene and substituted derivatives	1.55	0.00	1.75	down

MADN0421	C7H6O4	2,6-Dihydroxybenzoic acid	Organic acid And Its derivatives	1.27	0.01	1.13	down
MADN0432	C9H11NO4	L-Dopa	Amino acid and Its metabolomics	1.34	0.00	4.32	down
MADN0470	C45H79O13P	PI(36:4)	GP	1.07	0.08	1.84	down
MADP0068	C10H13N5O4	Deoxyguanosine	Nucleotide And Its metabolomics	1.49	0.03	1.84	down
MADP0147	C4H4N2	Pyrimidine	Nucleotide And Its metabolomics	1.72	0.00	2.61	down
MADP0191	C13H21N3O8S	S-Lactoylglutathione	Organic acid And Its derivatives	1.47	0.00	1.80	down
MADP0194	C7H8N2O2	N'-Methyl-2-pyridone-5-carboxamide	Heterocyclic compounds	1.23	0.01	1.14	down
MADP0264	C8H7NO2	Dioxindole	Heterocyclic compounds	1.57	0.00	1.68	down
MADP0267	C9H17NO4	Carnitine C2:0	FA	1.72	0.00	1.39	down
MADP0283	C7H11N3O	N-Acetylhistamine	Alcohols and amines	1.56	0.00	1.25	down
MADP0300	C9H17NO	TriacetonaMine	Heterocyclic compounds	1.03	0.09	1.99	down
MADP0311	C5H10N2O	3-Amino-2-piperidinone	Heterocyclic compounds	1.48	0.01	1.61	down
MADP0316	C11H15N3O6	N4-Acetylcytidine	Nucleotide And Its metabolomics	1.51	0.00	1.05	down
MADP0400	C8H16N2O4	Ser-Val	Amino acid and Its metabolomics	1.61	0.00	1.18	down
MADP0464	C32H66NO7P	PC(24:0)	GP	1.63	0.00	2.29	down
MEDN0041	C8H15NO3	Hexanoyl Glycine	Amino acid and Its metabolomics	1.58	0.01	3.07	down
MEDN0210	C6H12O5	1,5-Anhydro-D-Glucitol	Carbohydrates and Its metabolites	1.73	0.00	3.72	down
MEDN0350	C20H32O3	(±)15-HETE	FA	1.65	0.00	1.30	down
MEDN0531	C6H10O8	D-Glucarate	Carbohydrates and Its	1.59	0.00	1.35	down

			metabolites				
MEDN0570	C4H8O4	L-Erythrulose	Carbohydrates and Its metabolites	1.41	0.01	1.07	down
MEDN0750	C20H30O3	(±)12-HEPE	FA	1.59	0.00	3.04	down
MEDN0751	C20H32O3	(±)12-HETE	FA	1.68	0.00	2.38	down
MEDN0752	C20H30O3	(±)15-HEPE	FA	1.59	0.00	3.04	down
MEDN0753	C20H32O3	(±)16-HETE	FA	1.52	0.01	3.96	down
MEDN0755	C20H32O3	(±)17-HETE	FA	1.52	0.01	3.96	down
MEDN0756	C20H30O3	(±)18-HEPE	FA	1.59	0.00	3.04	down
MEDN0757	C20H32O3	(±)18-HETE	FA	1.52	0.01	3.96	down
MEDN0758	C22H32O3	(±)4-HDHA	FA	1.65	0.00	1.86	down
MEDN0760	C20H32O3	(±)5-HETE	FA	1.65	0.00	1.77	down
MEDN0763	C20H32O3	(±)9-HETE	FA	1.65	0.00	1.77	down
MEDN0765	C20H32O3	11,12-EET	FA	1.68	0.00	1.39	down
MEDN0769	C22H32O3	14(S)-HDHA	FA	1.69	0.00	3.44	down
MEDN0779	C20H34O3	5-HETrE	FA	1.57	0.00	1.46	down
MEDN0781	C20H34O6	6-keto-PGF1α	FA	1.62	0.00	1.74	down
MEDN0791	C20H34O5	PGD1	FA	1.61	0.00	1.20	down
MEDN0792	C20H34O5	PGE1	FA	1.61	0.00	1.20	down
MEDN0798	C20H32O5	PGK1	FA	1.51	0.01	1.58	down
MEDN1069	C18H28O2	FFA(18:4)	FA	1.37	0.02	1.34	down
MEDN1288	C20H35NO3	Glycine linoleate	GL	1.50	0.01	1.13	down
MEDN1314	C6H10O8	Mucic Acid	Carbohydrates and Its metabolites	1.59	0.00	1.35	down
MEDN1419	C20H30O3	12-OxoETE	FA	1.74	0.00	11.22	down
MEDN1421	C20H32O3	(±)8-HETE	FA	1.56	0.00	1.45	down
MEDN1422	C20H34O3	8(S)-HETrE	FA	1.57	0.00	1.46	down
MEDN1423	C20H34O3	15(S)-HETrE	FA	1.57	0.00	1.46	down
MEDN1430	C20H30O4	Prostaglandin A2	FA	1.54	0.00	1.55	down

MEDN1440	C22H32O3	13-HDoHE	FA	1.45	0.01	1.29	down
MEDN1441	C22H32O3	10-HDoHE	FA	1.47	0.00	1.60	down
MEDN1446	C20H36O3	11-HEDE	FA	1.66	0.00	1.68	down
MEDN1447	C20H36O3	15-HEDE	FA	1.66	0.00	1.68	down
MEDN1452	C20H32O5	11β-Prostaglandin E2	FA	1.55	0.00	1.00	down
MEDN1602	C10H13N5O4	3-Deoxyguanosine	Nucleotide And Its metabolomics	1.53	0.01	1.60	down
MEDN1684	C10H20N2O4	S-Ribosyl-L-homocysteine	Amino acid and Its metabolomics	1.56	0.00	1.21	down
MEDP0530	C20H28O3	15-deoxy-δ-12,14-PGJ2	FA	1.61	0.00	1.62	down
MEDP0898	C8H9NO3	Pyridoxal	Heterocyclic compounds	1.68	0.00	2.23	down
MEDP1177	C5H11NO2	N-Methyl-α-aminoisobutyric acid	Amino acid and Its metabolomics	1.50	0.00	1.02	down
MEDP1391	C25H45NO4	Carnitine C18:2	FA	1.61	0.00	1.19	down
MEDP1458	C22H30O2	FFA(22:7)	FA	1.68	0.00	1.54	down
MEDP1942	C6H11NO3	N-MethyTrans-4-Hydroxy-Proline	Amino acid and Its metabolomics	1.65	0.00	1.25	down
MEDP2024	C6H6O6	Dehydroascorbic acid	Organic acid And Its derivatives	1.35	0.01	1.37	down

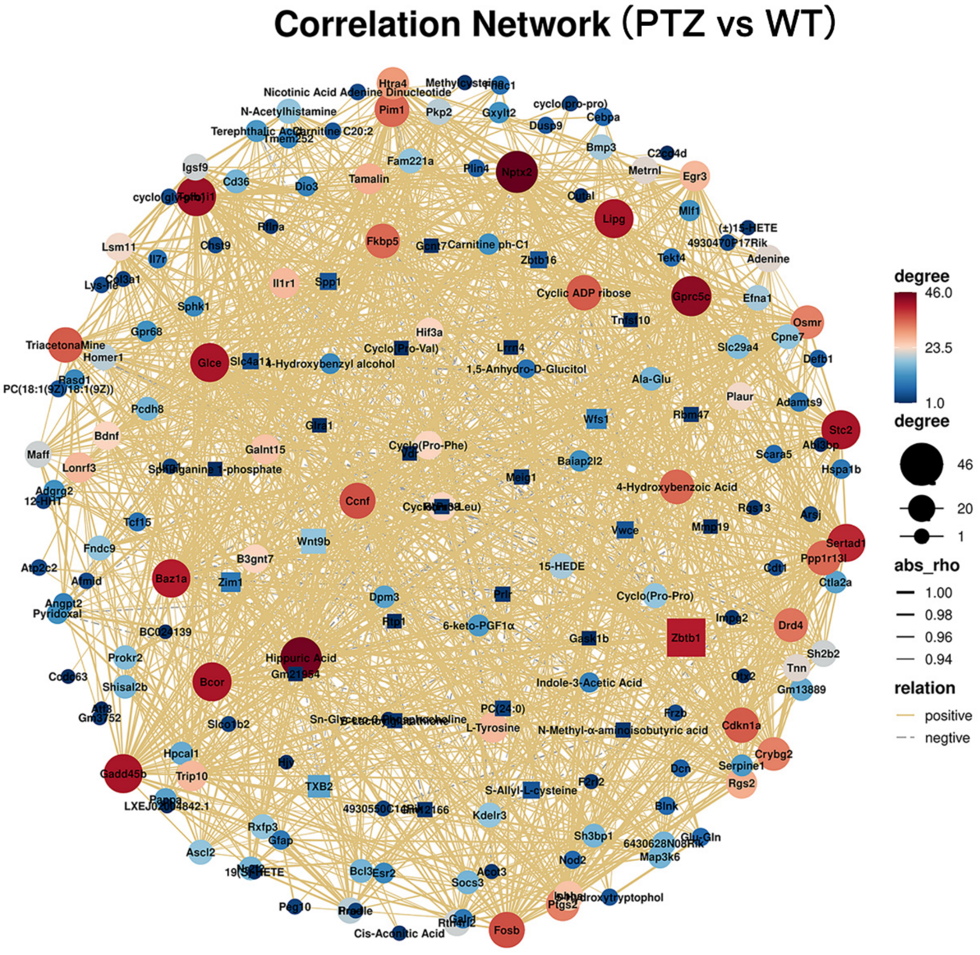

Figure S1. The correlation network between PTZ group and WT group.
